# Carbohydrate Counting as a Precision Method for Glycemic Control in Youth With Type 1 Diabetes: A Systematic Review and Meta‐Analysis

**DOI:** 10.1155/ijpe/5532465

**Published:** 2025-12-25

**Authors:** Poliana Viana Guarçoni, Luiz Gustavo Ribeiro Silva, Raquel Fonseca Sales, Tiago Ricardo Moreira

**Affiliations:** ^1^ Departamento de Medicina e Enfermagem, Universidade Federal de Viçosa, Viçosa, Minas Gerais, Brazil, ufv.br

**Keywords:** automated bolus calculator, carbohydrate counting, meta-analysis, systematic review, Type 1 diabetes mellitus

## Abstract

**Background:**

Although carbohydrate counting is a recommended method for managing diabetes, it is not widely used by patients with Type 1 diabetes mellitus. Aiming to emphasize the importance of this method, this study is aimed at quantitatively assessing the effectiveness of carbohydrate counting in reducing HbA1c levels in children and adolescents with Type 1 diabetes mellitus.

**Methods:**

Studies published between January 1993 and August 2024 were selected from the EMBASE, PubMed Central (PMC), Wiley Online Library, and ScienceDirect, without language restrictions. The primary outcome was to evaluate the reduction in HbA1c, comparing children and adolescents with Type 1 diabetes mellitus who did or did not use carbohydrate counting.

**Results:**

The results were synthesized through a meta‐analysis of the absolute mean difference in HbA1c between the groups. Seven studies were included in the meta‐analysis, involving a total of 599 individuals, with 276 in the control group and 323 in the intervention group. When compared to the control group, all carbohydrate counting methods resulted in a reduction in HbA1c. The variation ranged from −1.35% for the greatest reduction to −0.73% for the smallest reduction, with a final mean of −0.94% (95% CI: −1.13, −0.74). Subgroup analysis showed that the greatest reduction in HbA1c was observed in patients who were experienced in carbohydrate counting and used the automated bolus calculator.

**Conclusions:**

This study confirms the effectiveness of carbohydrate counting in reducing HbA1c levels in children and adolescents with Type 1 diabetes mellitus, reinforcing that even the basic form of this strategy represents a valuable and advantageous approach in the management of the disease.

## 1. Introduction

Type 1 diabetes mellitus (T1DM) is a chronic disease that affects millions of people worldwide, commonly diagnosed in childhood and adolescence [1]. Its main characteristic is the autoimmune destruction of pancreatic *β*‐cells, resulting in absolute insulin deficiency. Without proper treatment, T1DM can lead to severe complications, such as diabetic ketoacidosis (DKA) and long‐term microvascular and macrovascular complications. The American Diabetes Association (ADA) classifies diabetes as “a group of metabolic diseases characterized by hyperglycemia resulting from defects in insulin secretion, action or both,” within which T1DM stands out as the primary autoimmune form [2].

The vast majority of diabetes cases fall into two major categories: T1DM and Type 2 diabetes mellitus (T2DM) [3]. According to the International Diabetes Federation (IDF), it is estimated that 1,211,900 children and adolescents worldwide have T1DM, with approximately 149,500 new cases diagnosed each year [4]. T2DM will not be addressed in this study, which will focus on children and adolescents with T1DM.

The symptoms of T1DM, which are related to pronounced hyperglycemia, include polyuria, polydipsia, weight loss, and potentially polyphagia and blurred vision [2]. If a patient progresses with this condition, they may develop dehydration and acidosis, potentially leading to DKA [5].

The glycemic control of T1DM patients should be individualized, with variations depending on the clinical presentation. However, it is essential for patients to manage their condition via evaluation parameters such as glycated hemoglobin (HbA1c), capillary (or plasma) glucose levels, or time in range [6].

HbA1c is widely used to monitor diabetes control, as it reflects the average hemoglobin concentration in the blood over the past 8–12 weeks [7]. According to the Sociedade Brasileira de Diabetes, an HbA1c less than 7.0% is recommended for all individuals with diabetes to prevent microvascular complications (Class 1–Level A) and long‐term macrovascular complications (Class 1–Level B), provided that severe and frequent hypoglycemia does not occur [6].

T1DM patients should undergo insulin therapy to mimic physiological pancreatic secretion. The most common treatment method is the basal‐bolus insulin regimen, but continuous subcutaneous insulin infusion (insulin pump therapy) is also an option today [8]. In addition to insulin therapy, the treatment of T1DM includes blood glucose monitoring, physical activity, and nutritional therapy.

Carbohydrates are fully converted into glucose in the body, making them the macronutrients with the greatest impact on blood sugar levels. Therefore, managing carbohydrate intake plays a crucial role in blood glucose control and improves the prognosis for patients with diabetes [9]. In this context, one of the methods used for diabetes management is carbohydrate counting (CC), a strategy that has been recommended for over 20 years, particularly in the management of T1DM.

CC involves adjusting prandial insulin doses based on the estimated carbohydrate content of each meal. This approach offers a more flexible diet for people with diabetes by tailoring the insulin dosage to the amount of carbohydrates consumed. Moreover, by administering the appropriate amount of insulin, patients can maintain better control over their blood glucose levels, reducing the risk of complications like hypoglycemia and hyperglycemia [10].

However, despite its benefits, CC is often underutilized. This is due to several factors, such as the complexity of the process, insufficient training for both patients and healthcare providers, patient resistance (due to feeling overwhelmed by the number of calculations required at each meal), and lack of continuous support, which can discourage adherence to the method [11].

In practice, the method requires individuals to monitor pre‐ and postprandial blood glucose, calculate the carbohydrate intake, and adjust the insulin dose based on the preprandial glucose and the carbohydrates consumed. Thus, CC involves multiple steps during meals, including the use of tools like scales, calculators, nutrition labels, food measurement utensils, and smartphone apps. For this reason, it is essential for patients to have access to resources that can facilitate adherence to CC and ensure that the method is practiced effectively [10]. Consequently, accurately estimating the carbohydrate content of meals remains a challenge for patients, particularly children and adolescents, often leading to errors in bolus insulin administration [11].

CC can also be implemented at different levels of complexity. In this systematic review (SR), the following categories were considered: (1) basic CC, in which patients calculate prandial insulin doses without the support of digital tools; (2) experienced in CC, referring to patients with prior experience who can perform calculations accurately; (3) experienced in CC + ABC (automated bolus calculator), in which experienced patients use digital applications to facilitate insulin dose calculation; and (4) not experienced in CC + ABC, referring to patients without prior experience who rely on ABC applications for dose adjustments. Table 1 presents the type of intervention analyzed in each corresponding study, so that distinguishing between the levels of CC allows a better interpretation of differences in HbA1c reduction and clarifies the role of experience and technological support in improving adherence and glycemic control.

**Table 1:** Characteristics of the studies.

		**Number of individuals**											**Mean age (SD)**		**Diabetes duration (SD)**	
**Author, year**	**Country**	**Intervention**	**Control**	**Study type**	**Age range**	**Type of intervention**	**Definition of the intervention**	**Definition of the control**	**Insulin therapy (intervention)**	**Insulin therapy (control)**	**Study duration**	**Assessment of certainty of evidence**	**Control**	**Intervention**	**Control**	**Intervention**
Goksen et al., 2007 [12]	Turkey	14	24	Quasiexperimental	6–22 years	Basic carbohydrate counting	Carbohydrate counting associated with the use of insulin glargine.	Fixed insulin doses according to blood glucose levels.	Glargine, aspart, lispro, and Mixtard insulin	Glargine, aspart, lispro, and Mixtard insulin	6 months	83%	14.4 (3.9)	16.6 (3.3)	7.6 (4.5)	6.1 (3.9)
Dalsgaard et al., 2013 [13]	Brazil	93	93	Retrospective longitudinal study	5–18 years	Basic carbohydrate counting	Individualized meal plan with a predetermined amount of carbohydrates per meal and the possibility of exchanges between food groups, provided that the total carbohydrate amount is maintained.	Fixed insulin doses and a standardized diet with restriction of sweets and fixed caloric distribution.	—	—	6 and 12 months	100%	11.1 (2.66)	^b^	6.1 (3.2)	^b^
De Albuquerque et al., 2014 [14]	Brazil	14	14	Randomized clinical trial	10–19 years	Basic carbohydrate counting	Meal plan, daily log form, and home visits by nutritionists every 2 weeks to adjust the insulin‐to‐carbohydrate ratio.	Fixed insulin doses and a traditional meal plan based on exchanges, with phone calls from a dietitian every 2 weeks.	NPH, regular	NPH, regular	4 months	80%	14.28 (2.47)	13.7 (2.64)	^a^	^a^
Gökşen et al., 2014 [15]	Turkey	32	52	Randomized clinical trial	7–18 years	Basic carbohydrate counting	Training with a qualified team and use of the carbohydrate counting method to adjust insulin doses.	Traditional meal plan based on exchanges, with fixed insulin doses adjusted only according to blood glucose levels.	—	—	12 and 24 months	80%	17.9 (5.01)	16.44 (4.59)	8.97 (4.42)	8.08 (3.91)
Rabbone et al., 2014 [16]	Italy	23	62	Cohort	9–16 years	(1) Experienced in carbohydrate counting (2) Experienced in carbohydrate counting + ABC (3) Not experienced in carbohydrate counting + ABC	(1) Patients with previous experience in carbohydrate counting, using the technique since disease onset; without ABC. (2) Patients with previous experience in carbohydrate counting who also started using the ABC device for insulin bolus calculation. (3) Patients without previous experience in carbohydrate counting who were trained in CC at the beginning of the study and also received ABC.	Patients who declined to use carbohydrate counting (carbC) or the ABC device; no additional intervention with fixed insulin doses.	Long‐acting and rapid‐acting insulin analogs	Long‐acting and rapid‐acting insulin analogs	6 and 18 months	100%	13.1 (2.5)	12.1 (3.6)	4.3 (4.7)	12.1 (3.6)
Bayram et al., 2020 [17]	Turkey	27	26	Cohort	2–18 years	Adherents to basic and advanced carbohydrate counting	Structured training in advanced carbohydrate counting, including individualized insulin adjustments, detailed meal records, and both individual and group follow‐up, with subsequent evaluation and monitoring of correct adherence to the method.	After receiving the same training, nonadherent patients were those who showed a difference of ≥ 10 g between the carbohydrate estimate made by the patient and the dietitian’s assessment, patients who did not calculate the insulin dose correctly based on carbohydrate counting, or who did not use the insulin sensitivity factor (ISF) correctly.	—	—	6 months	100%	10.53 (3.71)	10.64 (4.40)	2.4 (0.29)	2.05 (0.48)
Sharma et al., 2023 [18]	India	64	61	Randomized clinical trial	6–18 years	Basic carbohydrate counting	Carbohydrate counting education with structured training (two sessions plus telephone follow‐up), including individualized insulin dose adjustment based on the insulin‐to‐carbohydrate ratio (ICR).	Routine clinical care with fixed insulin doses and an equally fixed dietary prescription, based on the child’s recommended nutritional requirements.	Insulin analogs and conventional insulin	Insulin analogs and conventional insulin	6 months	90%	11.3 (3.1)	11.4 (3.4)	3.5 (3.3)	4.4 (3.1)

Abbreviation: ABC = automated bolus calculator.

^a^Control: 0–5 years: 35.71%, 5–10 years: 42.86%, and 10–15 years: 21.43%. Intervention: 0–5 years: 71.43%, 5–10 years: 7.14%, and 10–15 years: 21.43%.

^b^These are the same individuals, but information on age and duration of diabetes was collected only during the intervention.

Therefore, this SR and meta‐analysis is aimed at evaluating whether CC is effective in reducing HbA1c in children and adolescents with T1DM. The most recent update on the topic was conducted in 2022 [19], with significant differences in both methodology and final results compared to the present study.

## 2. Methods

### 2.1. Data Source and Search Terms

This study follows the PICOT (Population, Intervention, Comparison, Outcome, and Type of study) framework for clinical questions. Accordingly, the present study is a SR that investigates, in children and adolescents aged 0–18 years diagnosed with T1DM (P), the effects of CC (I) compared to fixed‐dose insulin administration (C), with the primary outcome being the reduction of HbA1c (O).

The search strategy was based on the analysis of keywords used in relevant literature and previous reviews. Using the identified keywords, appropriate descriptors for the research were selected from Decs/Mesh (Health Sciences Descriptors) and Emtree.

On the basis of the identified descriptors, the search strategy was as follows: ((“Diabetes Mellitus Tipo 1” OR “Diabetes Mellitus, Type 1” OR “insulin‐dependent diabetes mellitus”) AND (“Contagem de carboidratos” OR “carbohydrate counting” OR “Dietary Carbohydrates” OR “Carboidratos da Dieta” OR “carbohydrate intake”) AND (Insulina OR Insulin) AND (Criança OR Child OR “Child, Preschool” OR Adolescente OR Adolescent)).

Owing to a limitation in the number of descriptors allowed in the ScienceDirect database, only English descriptors were used for that platform: ((“Diabetes Mellitus, Type 1” OR “insulin dependent diabetes mellitus”) AND (“carbohydrate counting” OR “Dietary Carbohydrates” OR “carbohydrate intake”) AND Insulin AND (Child OR “Child, Preschool” OR Adolescent)).

This SR included searches in electronic databases through the CAPES Journals Portal, comprising the following databases: Excerpta Medica dataBASE (EMBASE), PubMed Central (PMC), Wiley Online Library, and ScienceDirect (Elsevier), conducted in August 2024 to identify eligible studies. We analyzed all the references from the articles included in the study and used the artificial tool Litmap to identify articles related to the topic. We did not conduct searches in gray literature.

### 2.2. Eligibility Criteria

To be eligible for full‐text screening, articles had to meet all of the following criteria: (i) the population must include children and adolescents (under 18 years) diagnosed with T1DM; (ii) the study must include one group using CC as a method of insulin application; (iii) another group using preset insulin doses; (iv) the study must report changes in HbA1c between the two groups over a minimum interval of 3 months between intervention and outcome; (v) the study must be original; and (vi) the study design must be a clinical trial, quasiexperimental trial, or longitudinal study.

Only data published in peer‐reviewed journals were considered to ensure comparable study quality. Studies with the following characteristics were excluded: (i) application in T2DM; (ii) studies that did not provide HbA1c data; (iii) studies conducted before 1993; and (iv) studies involving patients using insulin pumps, as CC is essential for treatment in such cases, precluding comparisons with fixed‐dose insulin applications.

This date restriction was applied because CC was validated only by the ADA in 1993 [20]. Blinding of interventions between groups was not an inclusion criterion, as it is challenging to blind an intervention such as CC under the study conditions.

### 2.3. Study Selection

The title, abstract, and full‐text screenings were independently conducted by two reviewers (L.G.R.S. and R.F.S.), with disagreements resolved by a third reviewer (P.V.G.). Each of the two reviewers selected studies for potential inclusion on the basis of title and abstract content. Studies meeting the inclusion criteria were subsequently analyzed during the full‐text review.

### 2.4. Data Extraction and Outcomes

Data were extracted via the Rayyan–Intelligent Systematic Review application [21]. Two authors (L.G.R.S. and R.F.S.) extracted all the data, and both authors conducted a data accuracy analysis. The following data were collected: (i) title, authors, country, study duration, publication date, and study type; (ii) number of participants included, data collection instruments, and methods used for CC (intervention); and (iii) level of healthcare, characteristics of the included individuals, and key results of the intervention.

The following data were extracted from the results: (i) study follow‐up time; (ii) sample size of the control and intervention groups; and (iii) HbA1c values in both groups before and after the intervention. These outcome data were extracted for the baseline data and outcomes in the control and intervention groups for most studies. A spreadsheet was created in Microsoft Excel to extract all the information mentioned above.

### 2.5. Quality Assessment

The eligible studies were evaluated for quality via the assessment criteria from the Joanna Briggs Institute’s tool [12], which is specific to each study type: cohort, clinical, and quasiexperimental trials. Study quality was measured as a percentage, with points assigned to each item on the checklist as follows: 1 point for “YES,” 0.5 points for “unclear,” and 0 points for “NO.” Studies considered good quality were those scoring above 75%. The quality of the studies was not used as an exclusion criterion, but it was used to assess the methodological quality, identifying those that provide greater confidence in the findings. The risk of bias of the selected studies was assessed using the Newcastle–Ottawa Scale (NOS) assessment tool for observational studies (longitudinal and cohort) and Review Manager 5.4 (RevMan) for randomized clinical trials and quasiexperimental. The file with the values and results is in an additional file as supporting information at the end of the manuscript.

### 2.6. Data Synthesis

The meta‐analysis was conducted using a random‐effects model due to the heterogeneity found among the studies and absolute mean differences in HbA1c to calculate the effects in the control and intervention groups’ pre‐ and posttest samples, with the corresponding standard deviation, on improving clinical parameters across studies. Data from the HbA1c difference between the start and end of the intervention were used.

Heterogeneity was assessed via the chi‐square (*χ* ^2^) test with 90% significance (*p* < 0.10), and its magnitude was determined via the *I*‐squared (*I* ^2^) statistic [13]. Thus, heterogeneity was classified as low, moderate, or high when the *I* ^2^ values were above 25%, 50%, and 75%, respectively.

The meta‐analysis was conducted via Stata software (Version 11.0), and publication bias was examined via visual inspection of funnel plots and Egger’s test. The risk of bias was also analyzed via RevMan and NOS. An additional movie file provides more details (see Supporting Information). The statistical significance of the overall effect size of CC was determined by the 95% confidence interval (CI). Heterogeneity was explored via subgroup analyses or meta‐regression to investigate whether study‐level variables could explain the observed heterogeneity (type of study, type of intervention, and duration of intervention).

### 2.7. Protocol and Registration

This SR and meta‐analysis was conducted in accordance with the Preferred Reporting Items for Systematic Reviews and Meta‐Analyses (PRISMA) reporting guidelines [14] and was registered with the International Prospective Register of Systematic Reviews (PROSPERO) under the following protocol number: CRD42024555183.

## 3. Results

### 3.1. Study Selection

**Figure 1:**
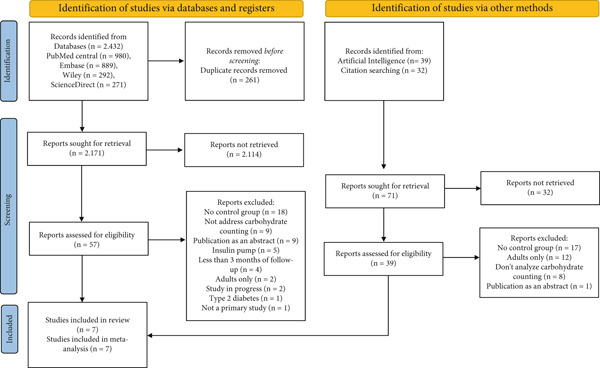
PRISMA flow diagram of study selection.

Figure 1 illustrates the process of the search and selection of articles. A total of 2432 studies were initially identified, of which 2171 remained after removing duplicates found across different databases. After screening the titles and abstracts, 57 studies were deemed eligible. Following a full‐text review, six studies met the eligibility criteria and were included in this SR [15–18, 22, 23]. In addition, one article [24] was incorporated through the Litmaps artificial intelligence tool, which uses one of the previously selected articles as a sentinel. No studies were included after the references of the accepted articles were reviewed. All seven studies selected for the SR were also included in the meta‐analysis.

### 3.2. Characteristics of the Studies

Among the included studies, three were conducted in Turkey [15, 17, 22], two in Brazil [16, 24], one in India [23], and one in Italy [18]. All studies were conducted at the outpatient level, and patient data were collected primarily through consultations and information available in medical records. The study designs were not uniform and consisted of three randomized clinical trials, one nonrandomized clinical trial, one retrospective longitudinal study, and two cohort studies. The age range of participants in the studies included in the meta‐analysis varied from 2 to 22 years. The studies were published between 2007 and 2023 and had varying intervention durations, with a minimum of 4 months and a maximum of 2 years.

In the intervention group, only two studies utilized both advanced and basic CC, whereas the remaining studies used only basic CC. The studies varied in terms of participant experience with CC and the use of accessory technologies, such as the ABC. In total, 276 individuals composed the control group, and 323 individuals composed the intervention group. As mentioned in the methods, studies were considered of good quality if they scored above 75% on the Joanna Briggs tools. Since all studies achieved scores above 80%, they were classified as high quality. Table 1 summarizes the main characteristics of the participants in the included studies.

### 3.3. Additional Outcomes

Overall, the included studies show important gaps regarding the assessment of additional outcomes beyond HbA1c. Severe hypoglycemia frequency was evaluated in three studies [15, 18, 22] and remained stable or slightly reduced, without a significant increase in events; mild or moderate hypoglycemia was rarely reported. Glycemic variability was assessed in two studies [16, 18], which demonstrated a reduction in hypoglycemia and hyperglycemia risk using the ABC, suggesting a potential benefit. Quality of life was evaluated in two studies [15, 23], showing improvement in the CC group, with reduced diabetes‐related worries and lower stress. Overall, while some evidence suggests benefits in quality of life and glycemic variability, the data remain limited, highlighting the need for future studies with systematic assessment of these outcomes.

### 3.4. Meta‐Analysis

**Figure 2:**
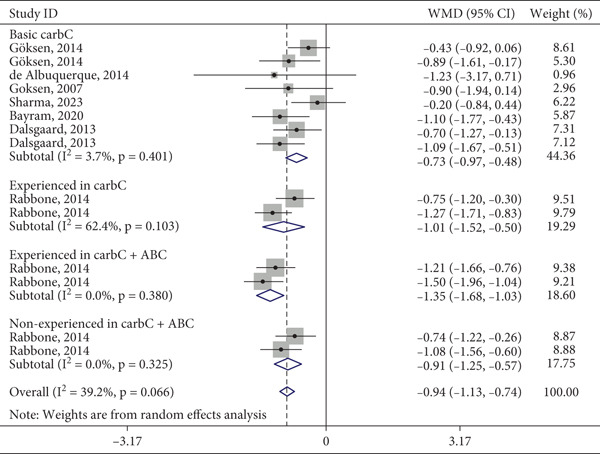
Forest plot of the difference in HbA1c by type of intervention.

The seven eligible articles were categorized according to the specific intervention type applied, as depicted in Figure 2. A primary focus across most studies was the introduction of CC to participants naïve to the technique; the sole exception was Rabbone et al. (2014). This particular study was unique in its design, incorporating multiple intervention arms: basic CC for beginners, advanced CC for experienced users, app‐assisted CC, and a non‐CC control group.

This multifaceted approach necessitates that Rabbone et al. (2014) be represented multiple times in Figure 2, corresponding to each distinct intervention arm. Conversely, other studies, which assessed only a single intervention, appear only once. Furthermore, the studies by Goksen et al. (2014), Dalsgaard et al. (2013), and Rabbone et al. (2014) are also listed twice in Figure 2, as they reported outcomes at two distinct time points: Goksen et al. (after 1 and 2 years), Dalsgaard et al. (after 6 months and 1 year), and Rabbone et al. (after 6 and 18 months). To elucidate this categorization schema and aid in the interpretation of Table 2, a summary of the intervention types used in each study is provided in Table 1.

**Table 2:** Distribution of the main characteristics of the studies according to intervention status.

	**Intervention**	**Control**	**Univariate metaregression**
	**Mean HbA1c (SD)**	**Mean HbA1c (SD)**	**p** **value**
Type of study			
Clinical trial	7.91 (1.59)	8.64 (1.78)	
Cohort	7.42 (0.56)	8.52 (1.06)	0.022
Retrospective	8.34 (1.70)	9.23 (2.24)	0.253
Type of intervention			
Basic carbC	7.98 (1.54)	8.80 (1.85)	
Experienced in carbC	7.47 (0.38)	8.48 (1.00)	0.265
Experienced in carbC + ABC	7.13 (0.45)	8.48 (1.00)	0.029
Nonexperienced in carbC + ABC	7.57 (0.65)	8.48 (1.00)	0.492
Duration of intervention			
4–6 months	7.83 (1.21)	8.68 (1.57)	
12 months	7.86 (1.31)	8.62 (1.76)	0.695
18–24 months	7.46 (0.73)	8.65 (1.18)	0.055

**Figure 3:**
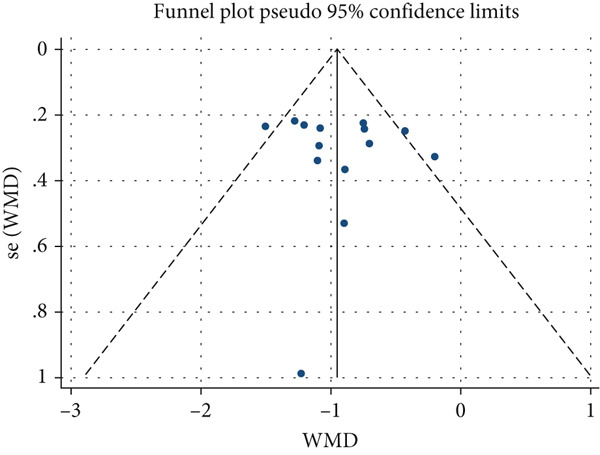
Funnel plot for symmetry assessment among studies.

All included studies were assessed for heterogeneity and publication bias. The analysis revealed moderate heterogeneity according to the *Q* test (*p* < 0.001) and *I* ^2^ statistic (*I* ^2^ = 39.2*%*). Figure 3 illustrates the symmetry among the investigations, confirmed by Egger’s test (*p* = 0.622), indicating a high likelihood that the SR captured and included smaller studies, which likely reported lower prevalences.

Figure 2 shows that all CC methods resulted in a reduction in HbA1c, with decreases ranging from −1.35% for the greatest reduction to −0.73% for the smallest, resulting in a final mean reduction of −0.94% (95% CI: −1.13, −0.74).

In the univariate analysis, the variables significantly associated with differences in HbA1c between the intervention and control groups (*p* < 0.1) were study type and type of intervention. The stratified model was constructed on the basis of the type of intervention, as it represents a better clinical parameter for evaluation. Additionally, heterogeneity decreased in three of the four analyzed subgroups. The duration of the intervention did not influence the study results. Table 2 presents the distribution of the main characteristics of the studies according to intervention status.

In the final model, the greatest reduction in HbA1c was observed in the group of patients experienced in CC who used an ABC, with a decrease of −1.35% (*p* = 0.380). This was followed by the method applied to individuals who were inexperienced in CC but also used the ABC, which resulted in a reduction of −1.01% (*p* = 0.103). The percentage of participants who experienced CC but did not use the ABC was −0.91% (*p* = 0.325). Finally, the group performing basic CC without prior experience and not using the ABC achieved a reduction of −0.73% (*p* = 0.401).

## 4. Discussions

The latest SR with meta‐analysis on CC was conducted in 2022 [25]. However, this review included only six studies, also considering the use of insulin pumps, which was not addressed in our study, as detailed in the methods. Only three studies were repeated between the reviews. Although other meta‐analyses have been conducted, they are older and do not include children and adolescents exclusively. For this reason, they have more articles [26, 27]. An updated review would allow a more in‐depth analysis of the effects of CC on the reduction of HbA1c, with new studies and considering the impact of new technologies, such as the ABC.

Our study demonstrated that CC, with or without the use of the ABC tool, is an effective method for glycemic control in individuals diagnosed with T1DM, yielding an average reduction of −0.94% in HbA1c compared with the traditional fixed‐dose insulin application method. Most of the included studies were randomized clinical trials and cohort studies, with significant data contributions from Turkey and Brazil. The analyzed data revealed that basic CC was the most commonly used intervention type and was present in five of the seven studies included in the SR. Regardless of the method employed, all types of interventions showed superior results in reducing HbA1c compared with the control groups.

According to the Standards of Medical Care in Diabetes, published in 2022 by the ADA, monitoring carbohydrate intake in the diet, as done through CC, is a fundamental strategy for glycemic control [28]. Our meta‐analysis reinforces the importance of this method in T1D treatment, given the significant reduction in HbA1c mentioned previously. These results align with findings from another study in the same field, which also reported a reduction in HbA1c in the group using CC compared with those using fixed insulin doses [25]. Similarly, a SR and meta‐analysis by Wiyono et al. revealed a reduction of −0.55% in HbA1c in the intervention group compared with the control group. As in our study, basic CC was the most prevalent method used in Wiyono et al.’s study [25].

In another SR and meta‐analysis, Fu et al. (2016) analyzed standard diets and diets with variability, such as low‐glycemic or personalized diets, compared to CC. They observed a significant reduction in HbA1c when CC was compared to conventional dietary education (SMD: −0.68, 95% CI: −0.98 to −0.38)—that is, when the control group followed a fixed diet without structured changes and with standardized insulin doses. On the other hand, when CC was compared to diets with modifications, such as low‐glycemic diets, the difference in HbA1c levels was minimal, with no substantial improvement (SMD: 0.02, 95% CI: −0.34 to 0.39). These findings suggest that the effect of CC on glycemic control is more evident when compared to traditional, rigid dietary strategies, tending to align with more individualized or structured dietary approaches. This is likely why we also observed significant reductions in HbA1c in our study, as we used fixed insulin doses as the control and, consequently, more restrictive diets.

The reduction in HbA1c found in our study also supports the recommendations of the SBD, which advises that individuals with diabetes mellitus using insulin should be instructed on dose administration on the basis of the amount of carbohydrates consumed. This approach is aimed not only at improving glycemic control but also at providing greater dietary flexibility (Class I, Level B) [29]. This objective is effectively achieved through CC, as it allows individuals to administer an ideal insulin dose for each meal. This personalized adjustment significantly reduces the occurrence of hypoglycemia or hyperglycemia episodes caused by inadequate insulin dosing. Furthermore, data aggregated from the Diabetes Control and Complications Trial (DCCT) and the United Kingdom Prospective Diabetes Study (UKPDS) show that maintaining HbA1c levels below 7% reduces both microvascular complications (e.g., retinopathy and nephropathy) and macrovascular complications (e.g., cardiovascular diseases), further emphasizing the importance of glycemic control [30, 31].

In the present study, CC methods that incorporated the use of the ABC tool achieved better results in reducing HbA1c. ABC is a technology that automatically suggests insulin doses on the basis of the carbohydrate content of the meal and the patient’s preprandial blood glucose levels. Users must input clinical data into the application, allowing ABC to perform the calculations [32]. The superior outcomes in HbA1c reduction can be attributed to the fact that using ABC minimizes errors in dose calculations, as the app performs calculations independently [33]. This benefit is significant, as it increases patient confidence in the implementation of the method. Another important factor associated with the method’s efficacy was the patient’s prior experience with CC. Familiarity with the method likely results in fewer errors when calculating carbohydrate amounts, fewer deviations related to meals, and greater ease in applying the method in challenging situations, such as parties or festive occasions.

Thus, CC should be recommended during medical consultations for patients with T1D, and the possibility of using ABC as a support tool should also be addressed. It is essential to guide patients in choosing safe applications recommended by the Food and Drug Administration (FDA) and SBD to ensure accuracy and safety in insulin dose calculations [32].

Additionally, diabetes education has proven to be critical for the efficacy of CC. Subgroup analysis revealed greater reductions in HbA1c in patients with experience in CC than in those without experience. These findings underscore that training in CC, combined with diabetes education, is indispensable for successful T1D management. This finding aligns with the guidelines of the Sociedade Brasileira de Pediatria (SBP), which state that “Diabetes education is the main tool for ensuring self‐care, enabling patients to achieve the self‐management required for adequate diabetes control” [34].

Two of the included studies involved participants over 18 years of age. However, owing to the sample size limitations of this SR, the authors decided to retain these studies and conduct a metaregression analysis to assess whether they could significantly influence the meta‐analysis. The metaregression results indicated that studies including participants over 18 years did not present significant differences compared with the other studies. Additionally, the mean ages of these studies fell within the parameters established for the SR and meta‐analysis. Therefore, these studies were included to increase the final sample size. Furthermore, this SR demonstrated that CC is effective even in older individuals, although this was not the primary focus of the research.

Furthermore, most of the included studies did not assess patient adherence to the intervention, a key determinant of the success of CC and a possible explanation for the heterogeneity observed in HbA1c reduction. Differences in adherence levels may have influenced the variability of outcomes across studies.

The Brazilian study by Uliana et al. [11] identified several factors associated with adherence to CC among adults with Type 1 diabetes, including higher family income, the use of advanced technologies for insulin administration and glucose monitoring, and regular follow‐up with endocrinologists and dietitians. Conversely, discontinuation of CC was associated with insufficient access to monitoring supplies and a lack of professional encouragement to maintain the practice. Practical barriers, such as limited time for calculations, difficulty in estimating carbohydrate content, and challenges in maintaining consistency during social events, were also cited as reasons for low adherence.

Therefore, the effectiveness of CC depends not only on the method itself but also on patient engagement, socioeconomic conditions, and continuous professional support. Future studies should systematically evaluate adherence levels to better interpret the magnitude of HbA1c reduction and the heterogeneity observed among interventions.

## 5. Limitations

This SR has several limitations, primarily related to methodological differences among the included studies. Specifically, two observational studies were included, which may reduce the robustness of the conclusions, as not all data originated from experimental studies.

Studies published between January 1993 and August 2024 were selected from EMBASE, PMC, Wiley Online Library, and ScienceDirect, without language restrictions. The search was extensive and complemented by manual screening of reference lists and previous SRs on the topic. Despite this comprehensive approach, only a limited number of randomized controlled trials were identified, which makes it difficult to draw firm conclusions and highlights the need for cautious interpretation of the findings. This limitation reflects the scarcity of experimental studies available in the literature, rather than a restriction of the search strategy.

Another limitation was the absence of complete data in some of the included studies. Variables such as diabetes duration, insulin doses, and insulin types were not consistently reported and therefore could not be analyzed. To address this issue, we contacted the corresponding authors but did not receive responses. This lack of qualitative data limited certain subgroup analyses.

Additionally, the studies did not evaluate patient adherence to the intervention, a critical factor for the success of CC. A recent Brazilian study [34] highlighted that adherence is influenced by factors such as support from endocrinologists and dietitians, adequate access to resources, and confidence in performing the required calculations. The efficacy of CC clearly depends on both patient adherence and external factors, such as professional support and infrastructure, which may affect the interpretation of the results related to nutritional management.

Moreover, HbA1c, although widely used as a glycemic control marker, has limitations. It can be influenced by nonglycemic factors such as anemia and hemoglobinopathies and does not accurately reflect daily glycemic fluctuations, particularly in patients with frequent hypoglycemia. Complementary methods, such as continuous glucose monitoring (CGM), provide a more detailed view of glycemic control. However, owing to its widespread acceptance, availability, and standardization in clinical guidelines, HbA1c was selected as the evaluation parameter in this SR. It remains the primary laboratory marker for diabetes control, playing a crucial role in assessing the risk of microvascular and macrovascular complications associated with the disease [35].

## 6. Conclusion

On the basis of the evidence presented in this SR and meta‐analysis, CC is an effective method for reducing HbA1c levels in children and adolescents with T1D. This technique proved superior to fixed‐dose insulin regimens in multiple daily injection (MDI) schemes, particularly when combined with tools such as the ABC, which demonstrated a greater impact on HbA1c reduction. The incorporation of ABCs can provide additional benefits in T1D management.

Although the included studies exhibited moderate heterogeneity, reflecting methodological and population variations, the results strengthen the case for the effectiveness of CC. The inclusion of smaller studies enhances the representativeness of the findings but underscores the need for future, more homogeneous, and rigorous research to validate and expand these results.

## Ethics Statement

The authors have nothing to report.

## Disclosure

All authors approved the final version submitted to the journal.

## Conflicts of Interest

The authors declare no conflicts of interest.

## Author Contributions

All authors contributed to the conception and design of the study and the analysis and interpretation of the data. P.V.G., L.G.R.S., and R.F.S. were involved in the literature review and manuscript drafting. L.G.R.S. and R.F.S. were responsible for data acquisition. T.R.M. conducted the critical and intellectual review of the manuscript.

## Funding

No funding was received for this manuscript.

## Supporting Information

Additional supporting information can be found online in the Supporting Information section.

## Supporting information


**Supporting Information 1** In the Supporting Information section, we provide a figure presenting the risk of bias assessment conducted using the RoB 2 (Risk of Bias 2) tool for the included studies. Additionally, a table is included detailing the application of the Newcastle–Ottawa Scale, along with a descriptive summary of the evaluated criteria and the corresponding scores.

**Supporting Information 2** We also provide the PRISMA 2020 checklist for systematic reviews, indicating the page number where each item is addressed in the main document.

## Data Availability

The data that support the findings of this study are available from the corresponding author upon reasonable request.
