# Supplementary material for: Carbohydrate Counting as a Precision Method for Glycemic Control in Youth With Type 1 Diabetes: A Systematic Review and Meta‐Analysis
Source: Int J Pediatr. 2025 Dec 25;2025:5532465. doi: 10.1155/ijpe/5532465 (PMC12752844; doi:10.1155/ijpe/5532465)
Supplement: Supplementary file 1 — Supporting Information 1 In the Supporting Information section, we provide a figure presenting the risk of bias assessment conducted using the RoB 2 (Risk of Bias 2) tool for the included studies. Additionally, a table is included detailing the application of the Newcastle–Ottawa Scale, along with a descriptive summary of the evaluated criteria and the corresponding scores. [file IJPE-2025-5532465-s001.docx]

**Article title**

Carbohydrate Counting as a Precision Method for Glycemic Control in Youth with Type 1 Diabetes: A Systematic Review and Meta-Analysis

**Risk of Bias**

**Rob2**

Fig. I: Risk of bias graph: review authors' judgements about each risk of bias item presented as percentages across randomized clinical trials and quasi-experimental included studies.


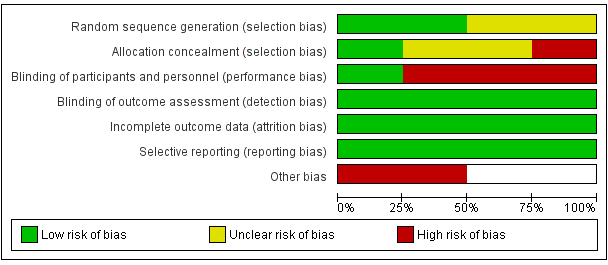


Other bias

**de Albuquerque, 2014** - A significant difference in baseline hemoglobin levels was observed between the control and intervention groups.

**Sharma, 2023** - No statistical test was applied; only the p-value was reported.

**Newcastle-Ottawa (NOS) scale**

The total score ranges from 0 to 9 stars, where:

Scores equal to or greater than 7 are considered high quality

Scores between 4 and 6 are considered high risk

Scores between 0 and 3 are considered very high risk of bias

Table I: Risk of bias graph: review authors' judgements about each risk of bias item presented as numbers across cohort and longitudinal included studies.


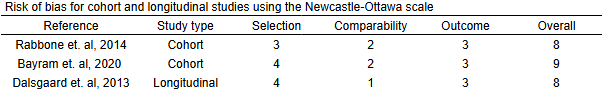


All included studies have more than 7 stars and are considered high quality.
